# Current treatment practice of functional abdominal pain disorders in children: A multicenter survey

**DOI:** 10.1007/s12664-022-01253-4

**Published:** 2022-09-03

**Authors:** Anouk M. Gorka, Femke Nauta, Merijn W. Bijlsma, Pieter Taselaar, Kay Diederen, Jeroen Hol, Nadia Oeij, Joery Goede, Maarten Rijpert, Gavin W. ten Tusscher, Frans B. Plötz

**Affiliations:** 1 Department of Pediatrics, Tergooi Hospitals, Rijksstraatweg 1, 1261 AN, Blaricum, The Netherlands; 2 Department of Pediatrics, BovenIJ Hospital, Amsterdam, The Netherlands; 3 Department of Pediatrics, Emma Children’s Hospital, Amsterdam UMC, University of Amsterdam, Amsterdam, The Netherlands; 4 Department of Pediatrics, Onze Lieve Vrouwe Gasthuis, Amsterdam, The Netherlands; 5 Department of Pediatrics, Flevo Hospital, Almere, The Netherlands; 6 Department of Pediatrics, Northwest Hospital Group, Alkmaar, The Netherlands; 7 Department of Pediatrics, Amstelland Hospital, Amstelveen, The Netherlands; 8 Department of Pediatrics, Spaarne Hospitals, Haarlem, The Netherlands; 9 Department of Pediatrics, Zaans Medical Center, Zaandam, The Netherlands; 10 Department of Pediatrics, Dijklander Hospital, Hoorn, The Netherlands; 11 Department of General Practice, Amsterdam University Medical Center, 1081 HV Amsterdam, The Netherlands

**Keywords:** Abdominal migraine, Children, Cognitive behavioral therapy, Functional abdominal pain disorder, Functional dyspepsia, Guideline adherence, Hypnotherapy, Irritable bowel syndrome, Pediatric, Rome III criteria

## Abstract

**Background:**

Approximately 90% of the children with chronic abdominal pain are diagnosed as having functional abdominal pain disorder (FAPD). The Dutch guideline “functional abdominal pain” provides a stepwise approach to treat FAPD. The aim of this survey was twofold first, to determine adherence to the Dutch guideline, and second to determine current management of FAPDs in clinical practice.

**Methods:**

A multicenter survey was designed. The survey was sent to pediatricians and pediatric residents in December 2020. The study ran from October 2020 until March 2021. Participants in ten hospitals in the western region of The Netherlands were invited to complete this survey. Respondents who indicated not to treat children with FAPDs or respondents who completed less than 3 steps of the survey were excluded.

**Results:**

In total, 85/174 (48.9%) respondents completed the survey. We included 80 respondents, 68 pediatricians and 12 pediatric residents, for analysis. Overall, self-reported guideline adherence was 85%. Self-reported adherence was higher than actual adherence. Only 50% of all respondents followed the first three steps of the guideline. The reported non-pharmacological and pharmacological treatments were diverse and varied between different age groups. The average follow-up duration was between 2 and 6 months, and the most regularly used outcome measures were attendance at school, quality of life, and adequate pain relief/reassurance.

**Conclusion:**

We reportedly observed a large variation in the management of children with FAPDs, due to low guideline adherence among clinicians. Improved guideline adherence may be accomplished by updating the guideline with specific recommendations per subtype, follow-up and outcome measures as well measures to improve guideline implementation.

**Supplementary Information:**

The online version contains supplementary material available at 10.1007/s12664-022-01253-4.

**Bullet points of the study highlights** ***What is already known?***

- There is a wide range of treatments for functional abdominal pain disorders (FAPDs).
- The Dutch Pediatric Society provides a guideline with stepwise approach to treat FAPDs, based on Rome III criteria.

***What is new in this study?***

- Self-reported adherence was higher than actual adherence.
- A reported large variability in management of children with FAPDs was observed.

***What are the future clinical and research implications of the study findings?***

- Improved guideline adherence may be accomplished by updating the guideline with specific recommendations per subtype, follow-up and outcome measures and measures to improve guideline implementation.

## Introduction

Chronic abdominal pain in children is one of the most frequent reasons to consult a pediatrician. Approximately 90% of the children with chronic abdominal pain are diagnosed as having functional abdominal pain disorder (FAPD), and in only 10% of the cases, a somatic cause is found [1]. Based on the Rome IV criteria, FAPDs can be classified into different subtypes, including functional dyspepsia, irritable bowel syndrome (IBS), abdominal migraine, and functional abdominal pain not otherwise specified [2, 3].

Treatments for FAPDs can be divided into pharmacological and non-pharmacological [4–6]. As for the pharmacological options, the effect of antispasmodic, antidepressant, anti-reflux, antihistaminic, and laxative agents on relieving FADP-related complaints was recently reviewed [6]. Some data suggested that peppermint oil, cyproheptadine, amitriptyline, famotidine, and polyethylene glycol are effective in children with FAPD although the overall quality of evidence was low. Additionally, a systematic review found some evidence that the probiotic Lactobacillus reuteri decreased the pain intensity in children with FAPD [7]. Several non-pharmacological therapies, like medical hypnotherapy, cognitive behavioral therapy (CBT), and probiotics, seem to show some beneficial effects [5, 8]. Furthermore, positive effects have been seen in patients treated with a diet containing low fermentable oligosaccharides, disaccharides, monosaccharides, and polyols (FODMAP) [4]. Additionally, non-pharmacological options are applied for which evidence is lacking, such as dietary interventions and complementary and alternative medicine [9].

The Dutch Pediatric Society published in 2015 a guideline “functional abdominal pain,” which provides a stepwise approach for the management of FAPDs [10]. However, the wide range of treatment options and the lack of clear evidence on treatment success rates, as well as a guideline that fails to provide unambiguous treatment procedures, may lead to a large variety of treatment practices. Hence, the aim of this survey was twofold; first to determine adherence to the Dutch guideline, and second to determine current management of FAPDs in clinical practice.

## Methods

### Study design and procedure

A multicenter survey study was designed. The survey was sent to pediatricians and pediatric residents in December 2020 and the entire study ran from October 2020 until March 2021.

### Participants

Participants in ten hospitals in the western region of The Netherlands were invited to complete this survey. Participating hospitals were part of the Pediatric Research and Evaluation Network (PREN) Amsterdam, which includes Tergooi Hospitals, Amsterdam UMC, BovenIJ Hospital, Amstelland Hospital, Noordwest Hospital Group, Onze Lieve Vrouwe Gasthuis (OLVG), Spaarne Hospital, Zaans Medical Center, Flevo Hospital, and Dijklander Hospital. In all the hospitals, a contact person was appointed. This person provided a list with the email addresses of all pediatricians and pediatric residents. Respondents who indicated not to treat children with FAPDs or respondents who completed less than 3 steps of the survey were excluded.

### Guideline

The Dutch guideline “functional abdominal pain” was published in 2015 and provides a stepwise approach to treat FAPD (Supplemental Fig. 1) [10]. The goal of treatment is to resume daily activities, such as going to school and engaging in extracurricular activities. Briefly, if the diagnosis of FAPDs is made according to Rome III classification, the first step in the guideline is to differentiate FAPDs into one of the five sub-classifications, namely abdominal migraine, functional dyspepsia, IBS, functional abdominal pain, and functional abdominal pain syndrome. The second step consists largely of reassurance and education about the condition to both parents and child. As part of the education, attention should also be paid to a healthy lifestyle, stress reduction, and nutrition. The third step is hypnotherapy or CBT if after 3 to 4 weeks symptoms persist. In addition to these steps, the guideline recommends in specific cases Lactobacillus GG for IBS and acid inhibition therapy for functional dyspepsia. Finally, in approximately one-third of the children, complaints persist in the long-term despite adequate explanation and reassurance, and in these children a number of pharmacological and non-pharmacological approaches can be considered by the treating physician. There is no specific recommendation, except laxatives for IBS with constipation and peppermint oil as an antispasmodic. The effects of these measures are evaluated after 2 to 4 weeks.

### Survey

The survey contained various questions concerning the management of children with chronic abdominal pain diagnosed as FAPD. The survey questions were divided into five parts. Part I contained questions related to the respondent, including specialization, years of experience, hospital type, classified as an academic or regional hospital, and the annual number of children with FAPDs treated by the respondent. Part II contained questions about initial FAPD management and whether this is the same as outlined by the guideline. Part III contained questions regarding familiarity with and use of the guideline. Respondents were asked in a step-by-step approach whether they apply a treatment according to the guideline or not. These questions were designed to address (self)-adherence to the guideline. Part IV contained specific questions about non-pharmacological and pharmacological therapies applied in FADPs, sub-classified according to Rome classification and if treatment strategy depends on subtype or age. By asking these questions, it was possible to get an impression about whether therapies are mainly used in a particular age group or for a certain subtype. Part V contained questions about follow-up and outcome measures. Most questions could be rated on a Likert scale with five categories: never, rarely, sometimes, regularly, and always. Depending on the answers given, the survey contained approximately 50–60 questions and took approximately 15 min to complete.

The survey was constructed in Castor EDC (Castor Electronic Data Capture, 2019), and sent by email. The invitation contained a unique link, which could only be used once. A reminder was sent after 9 days and after 4 weeks. In addition to the reminder emails, the contact persons in the hospitals were asked to stimulate pediatricians and pediatric residents to complete the survey.

### Data and statistical analysis

Guideline adherence was defined by following the first three steps, namely sub-classification of FAPD, explanation and reassurance, and medical hypnosis or CBT in chronological order. To measure guideline adherence, the answers “regularly” or “always” were considered in line with the guideline, contrary to “never,” “rarely,” or “sometimes.” Furthermore, the respondents, who self-reported that they used the guideline, were assessed if they actually followed the guideline steps correctly. To assess whether a type of treatment was used significantly more often in a FAPD subtype or age group, we performed Fisher’s exact test or Chi-square (*χ*^2^) test, as applicable. Bonferroni correction was made to correct for type I error caused by multiple testing. Data was exported and analyzed using the software RStudio (R version 4.0.3, RStudio version 1.4.1103, RStudio Team, 2021. RStudio: Integrated Development Environment for R. RStudio, PBC, Boston, MA, USA).

## Results

### Participants

**Fig. 1:**
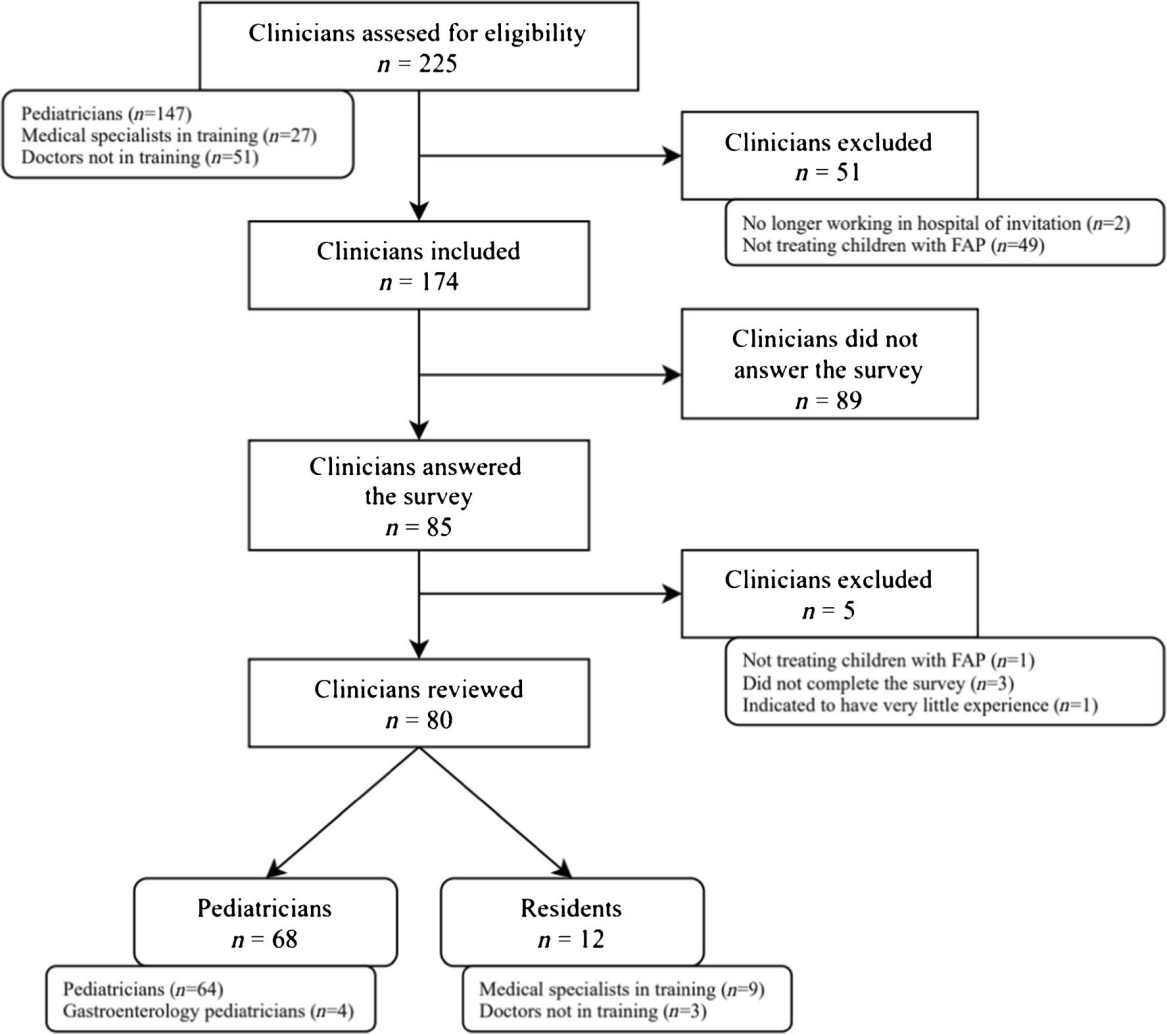
Study flowchart. *FAP* functional abdominal pain

The survey was sent to 225 pediatricians and pediatric residents, of whom 51 met the exclusion criteria (Fig. 1). The overall response rate of the survey was 85/174 (48.9%). Five surveys were excluded for various reasons (Fig. 1). We included 80 respondents, 68 pediatricians and 12 pediatric residents, for analysis. The baseline characteristics of the respondents are shown in Table 1. The majority of the respondents were pediatricians with more than 10 years of clinical experience.

**Table 1:** Baseline characteristics of respondents (*n*=80)

	Pediatricians (not-GE) *n* =64 (%)	Pediatricians (GE) *n*=4 (%)	Residents* n=12 (%)
*Type of hospital*			
Academic	8 (12.5)	3 (75.0)	4 (33.3)
Non-academic	56 (87.5)	1 (24.0)	8 (66.7)
*Years of experience*			
0–5 years	3 (4.7)		8 (66.7)
5–10 years	11 (17.2)		4 (33.3)
10–15 years	15 (23.4)	1 (25.0)	
>15 years	35 (54.7)	3 (75.0)	
*Yearly number of children with FAPDs*			
1–20 children yearly	25 (39.1)	1 (25.0)	8 (66.7)
20–50 children yearly	30 (46.9)		3 (25.0)
>50 children yearly	9 (14.0)	3 (75.0)	1 (8.3)
*Experience with managing child with FAPDs*			
Very experienced	21 (32.8)	3 (75.0)	3 (25.0)
Sometimes experiences difficulties	40 (62.5)	1 (25.0)	9 (75.0)
Often experiences difficulties	3 (4.7)		

*Residents, medical specialists in training and doctors not in training. *FAPD* functional abdominal pain disorder, *GE* gastroenterologist

### Guideline adherence

Sixty-eight (85%) of the 80 respondents claimed to adhere to the first three steps of the guideline recommendations. After analyzing if all initial three steps of the guidelines were followed, it appeared that self-reported adherence was higher than actual adherence. Only 50% of all respondents followed the first three steps of the guideline. Respondents who indicated using the guideline did not actually follow the guideline significantly better than the group who indicated they did not follow the guideline (Table 2).

**Table 2:** Respondents’ self-reported guideline adherence (*n* = 80)

Steps flowchart guideline	Respondents who indicate to use guideline *n*=68 (%)	Respondents who indicate not to use the guideline *n*=12 (%)	*p*-value	Bonferroni correction*
Step 1: Define subtype				
a. Yes b. Sometimes/specific	35 (51.5) 8 (11.8)	5 (41.7) 2 (16.7)	1.0000^F^	NS
Step 2a: Explanation and reassurance				
a. Healthy lifestyle b. Stress reduction c. Nutrition	41 (60.3) 37 (54.4) 35 (51.5)	6 (50.0) 5 (41.7) 5 (41.7)	0.5387^F^ 0.6159^C^ 0.7180^C^	NS NS NS
Step 3: Cognitive behavioral therapy or medical hypnosis	38 (55.9)	6 (50.0)	0.9498^C^	NS
Step 4a: Irritable bowel syndrome—probiotics	1 (1.5)	0 (0)	1.0000^F^	NS
Step 4b: Functional dyspepsia—acid inhibition	19 (27.9)	5 (41.7)	0.4949^F^	NS

*Bonferroni correction: *p*-values should be <0.00714 (0.05/7 tests) to hold statistical significance. *NS* not significant

^F^Fisher’s exact test

^C^Chi-squared test (*χ*^2^)

**Fig. 2:**
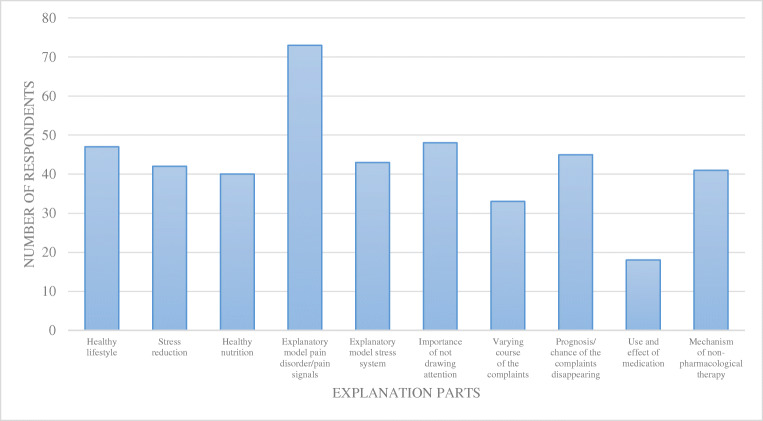
Step 2 guideline: explanation of functional abdominal pain disorder and reassurance

About half of the respondents indicated to use the FADP sub-classification. The second step of the guideline is to explain FADP and to reassure. Respondents paid particular attention to address explanation of the pain disorder/pain signals model, the importance of not drawing attention to the complaints, and a healthy lifestyle (Fig. 2). If symptoms persisted, then this was followed by non-pharmacological treatment by 53/80 (66.3%) of the respondents, with the majority opting for medical hypnosis, wait-and-see policy by 21/80 respondents (26.3%), and pharmacological treatment by 6/80 respondents (7.5%).

### Pharmacological and non-pharmacological treatments

Laxatives and peppermint oil were the most regularly reported pharmacological treatments (Table 3). As for the non-pharmacological treatments, psychological treatments (cognitive behavior therapy [CBT] and medical hypnosis) and nutritional advice were most commonly advised, which included trying fiber-rich food, followed by low FODMAP diet, trial treatment with lactose-free diet and other varied dietary options. Sub-analysis showed that pharmacological treatments were more often prescribed for children of 13–18 years of age, while these were prescribed to a lesser extent in children aged 4–7 years. No significant difference was found in the use of non-pharmacological treatments between the different age groups. Peppermint oil is mainly prescribed in IBS, and laxatives are mainly used in IBS and functional abdominal pain. Nutritional advice is mostly used for children with IBS.

**Table 3:** Pharmacological and non-pharmacological treatment specified per functional abdominal pain disorder subtype and age

	FD	IBS	AM	FAP	FAPS	Age 4–7	Age 8–12	Age 13–18
*Pharmacological treatment*								
Acid inhibition therapy (*n*=60)	32 (53.3)	1 (1.7)					5 (8.3)	10 (16.7)
Pain relief medication								
Paracetamol (*n*=34) Non-paracetamol (*n*=20	17 (50.0) 8 (40.0)	22 (64.7) 10 (50.0)	28 (82.4) 18 (90.0)	23 (67.6) 10 (50.0)	24 (70.6) 10 (50.0)	3 (8.8) 1 (5.0)	4 (11.8) 4 (20.0)	4 (11.8) 4 (20.0)
Antispasmodic agents								
Peppermint oil (*n*=61) Mebeverine (*n*=18)	23 (37.7) 10 (55.6)	48 (78.7) 16 (88.9)	15 (24.6) 8 (44.4)	37 (60.7) 13 (72.2)	27 (44.3) 13 (72.2)	4 (6.6) 1 (5.6)	12 (19.7) 2 (11.1)	14 (23.0) 3 (16.7)
Laxatives (*n*=73)	25 (34.2)	57 (78.1)	23 (31.5)	49 (67.1)	39 (53.4)	16 (21.9)	17 (23.3)	17 (23.3)
Anti-diarrheal (*n*=2)		2 (100)				1 (50.0)	2 (100)	2 (100)
Anti-emetics (*n*=23)	17 (73.9)	2 (8.7)	9 (39.1)	3 (13.0)	2 (8.7)	3 (13.0)	4 (17.4)	4 (17.4)
Antibiotics (*n*=0)								
Antimigraine (*n*=22)			22 (100)			1 (4.5)	4 (18.2)	6 (27.3)
Anti-histaminic (*n*=2)	2 (100)	2 (100)	2 (100)	2 (100)	2 (100)	1 (50.0)	1 (50.0)	1 (50.0)
Antidepressants (*n*=3)	1 (33.3)	1 (33.3)	3 (100)	1 (33.3)	2 (66.7)		1 (33.3)	1 (33.3)
*Non-pharmacological treatment*								
Probiotics (*n*=45)	1 (2.2)	4 (8.9)		1 (2.2)	2 (4.4)	3 (6.7)	3 (6.7)	3 (6.7)
Nutritional advices (*n*=69)	49 (71.0)	61 (88.4)	39 (56.5)	52 (75.4)	44 (63.8)	8 (11.6)	10 (14.5)	10 (14.5)
Complementary and alternative medicine (*n*=34)	23 (67.6)	25 (73.5)	22 (64.7)	28 (82.4)	27 (79.4)	3 (8.8)	6 (17.6)	10 (29.4)
*Psychological treatment*								
CBT or medical hypnosis* (*n*=32) CBT** (*n*=40) Medical hypnosis** (*n*=43)	1 (3.1) 1 (2.5) 1 (2.3)	4 (12.5) 2 (5.0) 4 (9.3)	1 (3.1) 1 (2.5) 1 (2.3)	7 (21.9) 3 (7.5) 6 (14.0)	4 (12.5) 3 (7.5) 4 (9.3)	3 (9.4) 2 (5.0) 3 (7.0)	6 (18.8) 4 (10.0) 5 (11.6)	6 (18.8) 6 (15.0) 2 (4.7)

*Respondent allows psychologist to choose cognitive behavioral therapy (CBT) or medical hypnosis

**Respondent chooses themselves or in consult with a psychologist

*FD* functional dyspepsia, *IBS* irritable bowel syndrome, *AM* abdominal migraine, *FAP* functional abdominal pain, *FAPS* functional abdominal pain syndrome

### Follow-up

**Fig. 3:**
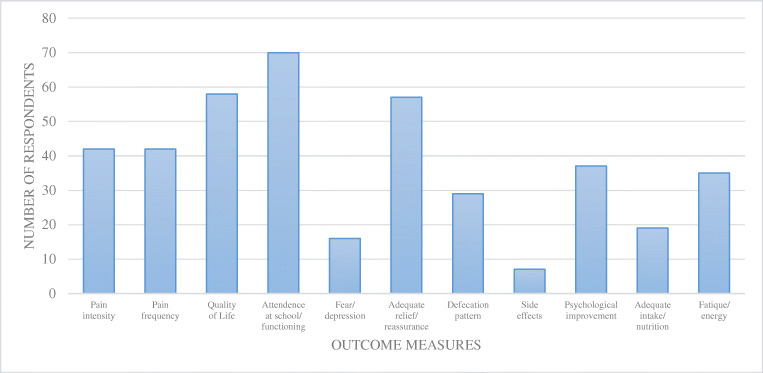
Outcome measures after treatment of functional abdominal pain disorders

In total, 41/77 (53.2%) of the respondents reported an average follow-up duration of 2–6 months, and 28/77 (36.4%) respondents claimed 6–12 months of follow-up. Treatment goals were regularly or always discussed by 47/77 (61.0%) of the respondents. The most frequently used treatment outcomes to evaluate the effect of the applied treatment, were attendance and functioning at school, quality of life, adequate relief, and reassurance (Fig. 3).

## Discussion

The results of our survey showed a large variation in the management of FAPDs among pediatricians and pediatric residents. We observed a discrepancy between self-reported and actual guideline adherence. Only 50% of all respondents followed the first three steps of the guideline. Additional non-pharmacological and pharmacological treatments were diverse and varied between different age groups. The average follow-up duration was between 2 and 6 months, and the most regularly used outcome measures were school attendance, quality of life, and adequate pain relief/reassurance.

In general, the development of evidence-based guidelines is an intensive and time-consuming process in which the weight of evidence for a treatment is systematically collected and assessed in a very careful and professional manner. Due to this process, update or renewal of guidelines may take considerable and unwarranted time. This is also true for the current guideline which was published in 2015 and based on the Rome III criteria. Shortly, thereafter the Rome IV criteria were published and the guideline has not been updated since this publication. Next to guideline development, it is also important to evaluate if a guideline is implemented in clinical practice. Developing a guideline is one thing, but implementing it and thus making it land properly in daily practice is another. If this process of implementation is not done properly, it can contribute to the variation in practice despite the existence of the good guideline.

The results of our survey demonstrate a low adherence to the current guideline. Low guideline adherence rates have been reported in pediatric clinical practice, highlighting the gap between the evidence-based and clinical practice [11–14]. Various reasons may contribute to this low adherence, which can be explained in terms of both the users and the guidelines. For example, Haskell et al. showed that dissemination of a clinical practice guideline is seldom sufficient to change practice and targeted interventions for behavior changes may improve compliance [12]. Also, ambiguity and large amount of criteria listed in guidelines may contribute to low guideline adherence [11, 13]. For this study, we did not explore in depth reasons why the guideline was not followed by the respondents. Respondents might indeed have determined their therapeutic strategy based on experience and encountered successes in the past. A possible guideline-related reason may be that defining subtypes of FAPDs seems more of a semantic discussion since the initial management steps in the guideline are the same for all subtypes, except for functional dyspepsia. Finally, although FAPD is a one of the most frequent reasons to consult a pediatrician, we showed previously that not all physicians regularly treat these children and hence are not familiar with the guideline [14].

Our survey showed that most respondents followed the step to start with hypnotherapy if symptoms persist after explanation and reassurance. The guideline does not distinguish between hypnotherapy or CBT for FAPDs because both treatments have been shown to be effective [15, 16]. The guideline recommends a number of treatments in four specific cases. Firstly, Lactobacillus GG is recommended for IBS. Our survey, however, shows that this recommendation is not followed by the respondents. An explanation for the lack of use of probiotics could be that it concerns non-reimbursed care or that respondents are not aware of the evidence and therefore do not use this treatment [17]. Secondly, acid inhibition therapy is recommended for functional dyspepsia, which is sometimes prescribed by the respondents. Remarkably, a recent systematic review indicated that pharmacological treatments were not recommended for functional dyspepsia [18]. Thirdly, laxatives are recommended for IBS with constipation. Laxatives are regularly prescribed by the respondents, especially for IBS and functional abdominal pain not otherwise specified. Fourthly, peppermint oil (antispasmodic) can also be considered in the treatment of children with FAPDs and was mainly prescribed in IBS [19]. Therapies that are not recommended in the guideline are generally not used by the respondents, with the exception of nutritional advice. Our survey showed that many respondents give nutritional advice in the treatment of IBS. Previous studies have shown light evidence for nutritional advice, though insufficient for recommendations [20]. The guideline does emphasize that time should be spent on discussing the importance of a healthy diet, but indicates that extra fiber intake is not recommended to purely improve symptoms.

The goal of treatment in FADP is to resume daily activities, such as going to school and engaging in extracurricular activities. It is remarkable that duration of follow-up and outcome measures are not described in the current guideline. Since we considered this an important issue when treating children with FAPD, we added a few questions regarding these topics in the survey. The most frequently used outcome measures were adequate relief and, or reassurance, followed by assumed quality of life, pain intensity, and frequency. A recent study advises measuring effectiveness using a core outcome set in trials, including pain intensity, pain frequency, quality of life, school attendance, anxiety/depression, adequate relief, defecation pattern (disease-specific for IBS), and adverse events [21].

We think that current guideline needs to be revised to increase adherence and to gain more evidence about management of FADPs in children. We therefore suggest a number of specific recommendations. First, the guideline should use the Rome IV diagnostic criteria for FAPDs and the classification. These classifications include functional dyspepsia, IBS, abdominal migraine, and functional abdominal pain not otherwise specified, respectively, and may be further sub-classified, for example IBS associated with constipation (IBS-C) or diarrhea (IBS-D). Second, we propose that for each (sub) classification of FAPDs, a strict therapeutic recommendation and a time-line are formulated. Drawback of the current guideline includes that the initial steps for all FAPDs are the same, i.e. explanation and reassurance, although some FAPDs may benefit from specific treatments. For example, in case of IBS-C, the start of laxatives medication could be part of initial therapy. Third, since the evidence base in children with FAPDs is small and many treatment suggestions are based on adult studies, we advocate that the guideline also formulate a uniform set of outcome measurements to evaluate the effect of therapy during follow-up [22].

We acknowledge that our survey comes with limitations. Firstly, the response rate was relatively low. This may be explained by the fact that all pediatricians and residents received an invitation. We did not primarily exclude pediatricians or residents who do not treat children with FAPDs. Secondly, since there is no information about the non-responders, there is a risk of underestimation and overestimation of the results. Finally, the Dutch guideline may not be applicable to other countries since measures, like CBT and hypnotherapy, are not performed by practicing pediatricians in other countries.

We reportedly observed a large variation in the management of children with FAPDs, due to low guideline adherence among clinicians. Improved guideline adherence may be accomplished by updating the guideline with specific recommendations per subtype, follow-up and outcome measures as well as measures to improve guideline implementation.

## Supplementary Information

Supplemental Figure 1. Flowchart Dutch guideline (PNG 213 kb)

## Declarations

### Disclaimer

The authors are solely responsible for the data and the contents of the paper. In no way, the Honorary Editor-in-Chief, Editorial Board Members, the Indian Society of Gastroenterology or the printer/publishers are responsible for the results/findings and content of this article.

## References

[1] Rajindrajith S, Zeevenhooven J, Devanarayana NM, Perera BJC, Benninga MA. Functional abdominal pain disorders in children. Expert Rev Gastroenterol Hepatol. 2018;12:369–90. 10.1080/17474124.2018.1438188 29406791

[2] Benninga MA, Faure C, Hyman PE, St James Roberts I, Schechter NL, Nurko S. Childhood functional gastrointestinal disorders: neonate/toddler. Gastroenterology. 2016;S0016-5085(16):00182–7. 10.1053/j.gastro.2016.02.016 27144631

[3] Hyams JS, Di Lorenzo C, Saps M, Shulman RJ, Staiano A, van Tilburg M. Functional disorders: children and adolescents. Gastroenterology. 2016;S0016-5085(16):00181–5. 10.1053/j.gastro.2016.02.015 27144632

[4] Korterink J, Devanarayana NM, Rajindrajith S, et al. Childhood functional abdominal pain: mechanisms and management. Nat Rev Gastroenterol Hepatol. 2015;12:159–71. 10.1038/nrgastro.2015.21 25666642

[5] Rutten JM, Korterink JJ, Venmans LM, Benninga MA, Tabbers MM. Nonpharmacologic treatment of functional abdominal pain disorders: a systematic review. Pediatrics. 2015;135:522–35. 10.1542/peds.2014-2123 25667239

[6] Korterink JJ, Rutten JM, Venmans L, Benninga MA, Tabbers MM. Pharmacologic treatment in pediatric functional abdominal pain disorders: a systematic review. J Pediatr. 2015;166:424-31.e6. 10.1016/j.jpeds.2014.09.067 25449223

[7] Trivić I, Niseteo T, Jadrešin O, Hojsak I. Use of probiotics in the treatment of functional abdominal pain in children-systematic review and meta-analysis. Eur J Pediatr. 2021;180:339–51. 10.1007/s00431-020-03809-y 32940743

[8] Vlieger AM, Menko-Frankenhuis C, Wolfkamp SC, Tromp E, Benninga MA. Hypnotherapy for children with functional abdominal pain or irritable bowel syndrome: a randomized controlled trial. Gastroenterology. 2007;133:1430-6. 10.1053/j.gastro.2007.08.072 17919634

[9] Vlieger AM, Blink M, Tromp E, Benninga MA. Use of complementary and alternative medicine by pediatric patients with functional and organic gastrointestinal diseases: results from a multicenter survey. Pediatrics. 2008;122:e446–51. 10.1542/peds.2008-0266 18662934

[10] Tabbers MM.. Richtlijn Functionele buikpijn bij kinderen. 2015. https://nvk.nl/themas/kwaliteit/richtlijnen/richtlijn?componentid=6881285&tagtitles=Maag-Darm-Leverziekten+(MDL),Sociale+en+Psychosociale+kindergeneeskunde Accessed May 2015

[11] Niele N, van Houten MA, Boersma B, et al. Multi-centre study found that strict adherence to guidelines led to computed tomography scans being overused in children with minor head injuries. Acta Paediatr. 2019;108:1695–1703. 10.1111/apa.14742 30721540

[12] Haskell L, Tavender EJ, Wilson CL, et al. Effectiveness of targeted interventions on treatment of infants with bronchiolitis: a randomized clinical trial. JAMA Pediatr. 2021;175:797–806. 10.1001/jamapediatrics.2021.0295 PMC8042564 33843971

[13] van der Weijden BM, Achten NB, Bekhof J, et al. Multicentre study found that adherence to national antibiotic recommendations for neonatal early-onset sepsis was low. Acta Paediatr. 2021;110:791–8. 10.1111/apa.15488 PMC7984438 32686180

[14] van Kalleveen MW, Noordhuis EJ, Lasham C, Plotz FB. Large variation in clinical practice amongst pediatricians in treating children with recurrent abdominal pain. Pediatr Gastroenterol Hepatol Nutr. 2019;22:225–32. 10.5223/pghn.2019.22.3.225 PMC6506428 31110955

[15] Vlieger AM, Rutten JM, Govers AM, Frankenhuis C, Benninga MA. Long-term follow-up of gut-directed hypnotherapy vs. standard care in children with functional abdominal pain or irritable bowel syndrome. Am J Gastroenterol. 2012;107:627–31. 10.1038/ajg.2011.487 22310221

[16] Abbott RA, Martin AE, Newlove-Delgado TV, et al. Psychosocial interventions for recurrent abdominal pain in childhood. Cochrane Database Syst Rev. 2017;1:CD010971. 10.1002/14651858.CD010971.pub2 PMC6464036 28072460

[17] Newlove-Delgado TV, Martin AE, Abbott RA, et al. Dietary interventions for recurrent abdominal pain in childhood. Cochrane Database Syst Rev. 2017;3:CD010972. 10.1002/14651858.CD010972.pub2 PMC6464236 28334433

[18] Browne PD, Nagelkerke SCJ, van Etten-Jamaludin FS, Benninga MA, Tabbers MM. Pharmacological treatments for functional nausea and functional dyspepsia in children: a systematic review. Expert Rev Clin Pharmacol. 2015;11:1195–208. 10.1080/17512433.2018.1540298 30360666

[19] Chumpitazi BP, Kearns GL, Shulman RJ. Review article: the physiological effects and safety of peppermint oil and its efficacy in irritable bowel syndrome and other functional disorders. Aliment Pharmacol Ther. 2018;47:738–52. 10.1111/apt.14519 PMC5814329 29372567

[20] Axelrod CH, Saps M. The role of fiber in the treatment of functional gastrointestinal disorders in children. Nutrients. 2018;10:1650. 10.3390/nu10111650 PMC6267171 30400292

[21] Zeevenhooven J, Rexwinkel R, Van Berge Henegouwen VWA, et al. Consensus Group on Outcome Measures Made in Pediatric Enteral Nutrition Clinical Trials Working Group. A core outcome set for clinical trials in pediatric functional abdominal pain disorders. J Pediatr. 2020;221:115–22. 10.1016/j.jpeds.2020.02.032 32312551

[22] Thapar N, Benninga MA, Crowell MD, et al. Paediatric functional abdominal pain disorders. Nat Rev Dis Primers. 2020;6:89. 10.1038/s41572-020-00222-5 33154368

