# Supplementary figures and images for: Current treatment practice of functional abdominal pain disorders in children: A multicenter survey
Source: Indian J Gastroenterol. 2022 Sep 3;41(4):369–77. doi: 10.1007/s12664-022-01253-4 (PMC9529717; doi:10.1007/s12664-022-01253-4)

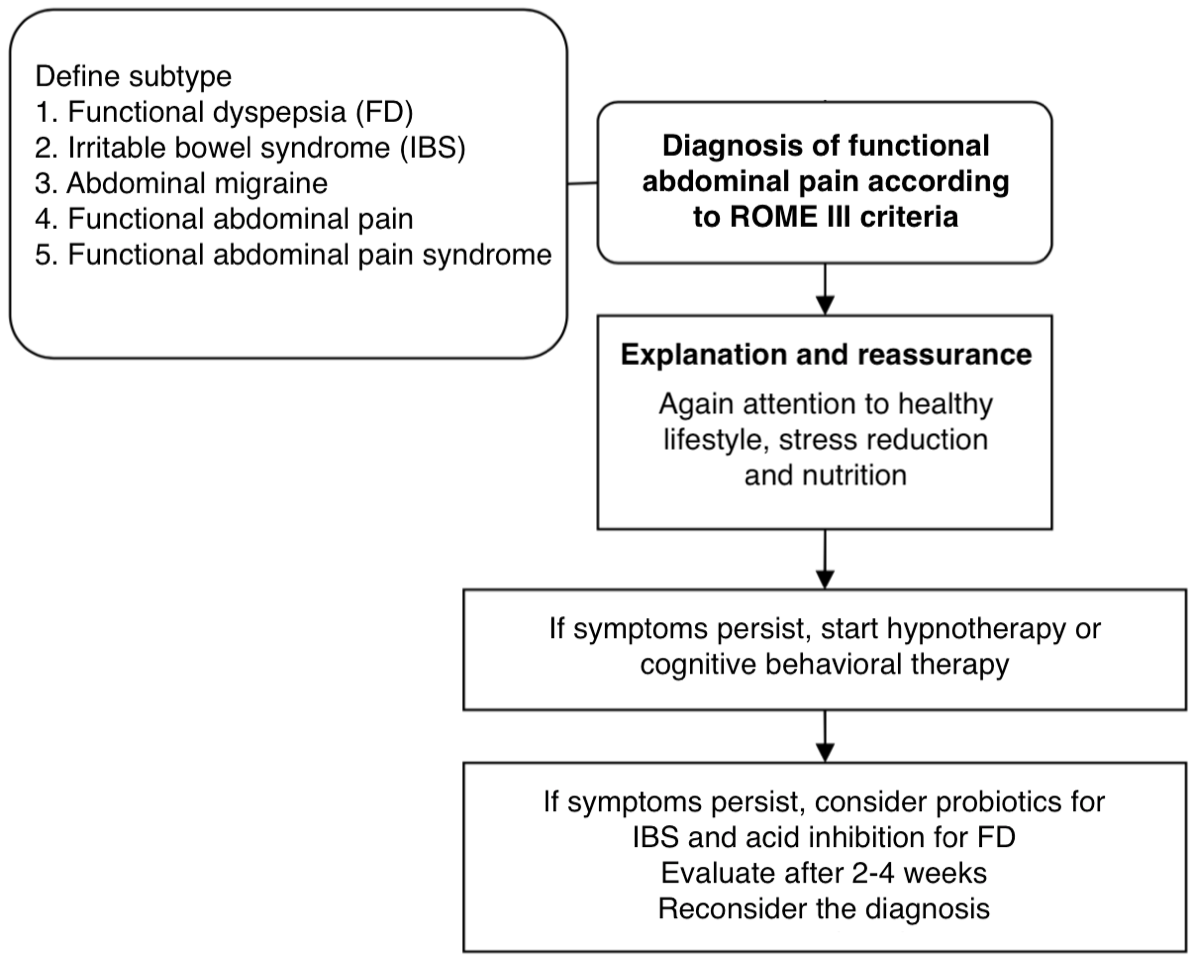

Supplement: Supplementary file 1 — Flowchart Dutch guideline (PNG 213 kb) [file 12664_2022_1253_MOESM1_ESM.png]
